# Family Dynamics and Personal Strengths among Dementia Caregivers in Argentina

**DOI:** 10.1155/2016/2386728

**Published:** 2016-06-20

**Authors:** Aaliah G. Elnasseh, Michael A. Trujillo, Silvina Victoria Peralta, Miriam E. Stolfi, Eliana Morelli, Paul B. Perrin, Juan Carlos Arango-Lasprilla

**Affiliations:** ^1^Virginia Commonwealth University, 806 W. Franklin Street, P.O. Box 842018, Richmond, VA 23284, USA; ^2^Instituto San Lucas Neurociencias, 1655 Paraguay, Santa Fe, Argentina; ^3^Virginia Commonwealth University, 800 W. Franklin Street, P.O. Box 842018, Richmond, VA 23284, USA; ^4^BioCruces Health Research Institute, Cruces University Hospital, Barakaldo, Spain; ^5^Basque Foundation for Science (IKERBASQUE), Bilbao, Spain

## Abstract

This study examined whether healthier family dynamics were associated with higher personal strengths of resilience, sense of coherence, and optimism among dementia caregivers in Argentina. Caregivers are usually required to assist individuals with dementia, and family members have typically fulfilled that role. Personal strengths such as resilience, sense of coherence, and optimism have been shown to protect caregivers from some of the negative experiences of providing care, though the family-related variables associated with these personal strengths are largely unknown. Hierarchical multiple regressions investigated the extent to which family dynamics variables are associated with each of the caregiver personal strengths after controlling for demographic and caregiver characteristics. A sample of 105 caregivers from Argentina completed a set of questionnaires during a neurologist visit. Family dynamics explained 32% of the variance in resilience and 39% of the variance in sense of coherence. Greater family empathy and decreased family problems were uniquely associated with higher resilience. Greater communication and decreased family problems were uniquely associated with higher sense of coherence. Optimism was not found to be significantly associated with family dynamics. These results suggest that caregiver intervention research focused on the family may help improve caregiver personal strengths in Argentina and other Latin American countries.

## 1. Introduction

Every year, approximately 7.7 million individuals develop dementia or Alzheimer's disease, a number that translates to one new case every 4 seconds [1]. This figure is expected to increase as assessments and diagnostic criteria improve [2] and as life expectancies continue to increase [3]. Dementia affects more people in Latin America (8.5%) than it does in the United States (6.5%) and Western Europe (6.9%) [4], and since dementia is more prevalent among older adults, the regions expected to be most heavily affected by the disease are the regions with a projected population increase in this age demographic [5]. Countries in Latin America and the Caribbean are expected to experience huge increases in the population group of individuals aged 60 and older in the next 40 years; the number of older adults is expected to quadruple in this time period, reaching about 186 million [3], with older adults outnumbering the young population by approximately 30% [6].

Dementia is a syndrome that affects several areas of the brain and leads to a decrease in cognitive functioning—usually characterized by progressive deceleration of brain function over time [7]. The primary symptoms of dementia include impairments in memory, language and/or communication difficulties, visual perception problems, a decreased ability to focus or pay attention, difficulties in planning or problem solving, and changes in mood and personality [7]. Short-term memory problems, especially, make it difficult for people with dementia to carry out everyday activities on their own [8]. As a result of the progressive nature of the disease, the assistance of informal caregivers typically becomes necessary.

Dementia caregiving is usually the responsibility of the spouse of an individual with dementia or an adult child [9]. Caring for aging adults with dementia is associated with increases in burden, distress, and decrements in mental health and well-being [10]. Studies generally suggest that caregiving for individuals with dementia is more stressful than caregiving for individuals with many other diseases [11]. This is because dementia caregiving is characterized by specific problems such as the lack of free time, isolation from others, behavioral problems and personality changes, and fewer positive experiences resulting from the lack of expressed gratitude by the care recipient [12].

Given that the vast majority of individuals with dementia are assisted by family members or other informal caregivers, it is important to consider the role of family dynamics in the caregiving experience. Family communication [13], adaptability/flexibility [14], and marital cohesion [15], for example, have all been connected to the emotional functioning of caregivers. Research utilizing a structural family framework has shown that family functioning plays a significant role in the stress processes of dementia caregivers [16]. Depression and anxiety, for instance, are more likely to occur among caregivers in families with poor functioning [17], and conflicted family dynamics can intensify caregiver depressive symptoms [18] and caregiver strain [19]. Similarly, conflict related to care issues may impact caregiver burden [20]. The poor functioning of families is also likely to result in a decrease in the time spent on patient care [21], potentially impacting the quality of care the individual with dementia receives. Conversely, healthier family dynamics, such as family support, are associated with lower levels of caregiver strain [22]. For example, when families give more support to primary caregivers, they often provide more help to the individual with dementia [21]. Caregivers experience less burden and depression when family cohesion is high [23], and greater family communication also plays an important role in reducing caregiver burden [24].

When caring for a family member with dementia, culture and ethnicity play a role in the caregiver's stress and coping processes. According to the sociocultural stress and coping model, culture influences the perception of social support and the appraisal of stressors, which may impact the well-being of caregivers [25–27]. Latino cultures in particular place more emphasis on the family with the cultural value of* familismo*, defined as strong identification and attachment of individuals to their nuclear and extended families and feelings of loyalty, reciprocity, and solidarity among family members [28], having an important role.* Familismo* values encourage the reliance on family members for support, feeling the obligation to care for family in need, and the reliance on relatives to provide guidance on life [28, 29]. Because Latinos report stronger commitment to their families [30], they may have different caregiving experiences compared to caregivers in more individualistic cultures. Additionally,* familismo* can influence caregiving experiences [31]. For example, Latino dementia caregivers are expected to avoid placing their family members in nursing homes or adult day care [30]. Since family-centered care is highly favored in Latin America [32], caregivers who consider placing a relative at such facilities often experience feelings of guilt and shame [33]. Subsequently, informal caregivers in Latin America spend more time caregiving compared to other racial/ethnic groups and experience poorer mental health outcomes [30]. Additionally, the culture of* familismo* influences the emotional distress experienced by Latino dementia caregivers through dysfunctional thoughts [34]. These dysfunctional thoughts are generally related to depressive symptoms [35] and, among Alzheimer's caregivers, to poor health outcomes [36].

Consistent with the sociocultural stress and coping model, caregivers of people with dementia appraise stressors in a variety of ways that may also aid in buffering the negative effects of caregiver burden. For instance, the literature identifies resilience, optimism, and sense of coherence as three of the most important personal strengths exhibited by caregivers that assist in adaptation to caregiver stress [37, 38]. Research on caregiver resilience shows that it is more related to caregiver variables than to situational variables [37]. Similarly, optimism [39] and sense of coherence [40] are caregiver variables related to coping adaptations. As such, all three personal strengths are expected to help buffer the negative effects of caregiver burden. These personal strengths are also of interest to family caregivers as research has shown that family functioning influences the development of coping strategies such as resilience and sense of coherence [41]. And in the context of family dementia caregivers, optimism has been related to caregiver burden [42], health, and feelings of anger [43]. As such, the impact of family dynamics on a caregiver's adaptation to stress may be especially relevant for Latino caregivers as the role of familism is likely to affect their coping style and personal strengths.

The first important personal strength for caregivers is resilience. Resilience is a psychological phenomenon characterized by effective coping and adaptation in the face of loss, hardship, or adversity. In the context of dementia, resilience is believed to be a protective factor for caregiver stress [44] and is related to lower levels of depression [45] and better emotional and physical health outcomes for caregivers [37]. Higher levels of perceived control and the belief that life's challenges are opportunities to increase skills and self-knowledge are some characteristics associated with increased resilience [45]. High levels of social support, especially from within the family, are also associated with higher resilience [44]. Resultantly, these characteristics can lead caregivers to experience “uplifts” in caregiving [11].

Optimism is a personality trait characterized by a disposition to expect positive outcomes in the face of adversity and hardship. Optimism is associated with more positive affect, less negative affect, and better mental health [46]. Caregivers high in trait optimism view their own coping behaviors more positively and are more likely to perceive their coping strategies as effective [46]. Optimism is associated with decreased stress and fewer symptoms of depression [47], and optimistic caregivers experience more positive affect, less negative affect, and better mental health [47]. Optimism is also shown to be related to greater satisfaction in martial and child-parent relationships and healthier family communication [48, 49].

Sense of coherence is a personality trait that includes a set of positive coping strategies which enable people to use available resources efficiently [50] and has often been used in the research literature interchangeably with the term “coping.” Sense of coherence is composed of three factors: comprehensibility (the feeling that the world makes sense and that information regarding the environment is ordered, consistent, and explainable), manageability (the feeling that adequate resources are available for meeting internal and external demands), and meaningfulness (the feeling that these internal and external demands deserve investment and engagement) [50, 51]. Higher sense of coherence benefits dementia caregivers in many ways and has been consistently associated with lower burden, anxiety, and depression [50]. Within the context of the family, greater sense of coherence is related to increased affection and easier communication [52]. The relation between depressive symptoms and caregiver strain has been found to be mediated by sense of coherence [38]. Higher sense of coherence is thus characterized by a salutogenic (promoting health) orientation toward stressors [53].

Although Latin America is expected to experience increases in dementia rates, not enough research has been conducted on dementia caregivers in the region. The existing research has primarily operated from a deficit model examining the negative aspects of care such as burden, depression, and other psychosocial problems in caregivers. Furthermore, very little research has examined particular cultural strengths that may aid in dementia caregiving in Latin America [37], and no studies have been conducted on the relationship between family dynamics and personal strengths in this population—despite the research documenting the importance of family values in this population. The purpose of this study is to examine whether healthier family dynamics are associated with higher sense of coherence, resilience, and optimism in dementia caregivers in Latin America. It is hypothesized that healthier family dynamics will be related to a higher sense of coherence, greater resilience, and more optimism. From the research on the significance of family functioning in reducing caregiver burden [19, 20, 22, 23], family pathology and cohesion are hypothesized to be uniquely related to greater resilience, sense of coherence, and optimism. Consistent with prior work [24], greater family communication is also hypothesized to be uniquely associated with resilience, sense of coherence, and optimism. Finally, empathy, a factor associated with social support, is also hypothesized be related to resilience and sense of coherence.

## 2. Method

### 2.1. Participants

Caregivers of individuals with dementia (*N* = 105) from Argentina participated in this study. For a summary of caregiver demographics see Table 1. Recruitment of participants was from the Instituto de Neurosciencias de San Lucas in Rosario, Argentina. Caregivers were identified as individuals caring daily for an individual with dementia, and the inclusion criteria were as follows: being at least 18 years of age, identifying as primary caregiver of the person with dementia, providing care for a minimum of three months, being well-informed about the patient's medical and family history, and not having a history of serious psychiatric or neurological disorders. For all participants, informed consent was provided and the data were collected according to the Institutional Review Board approval at the University of Deusto.

### 2.2. Measures

A questionnaire was created to collect demographic information from caregivers. Caregiver personal strengths and family dynamics were assessed using a series of questionnaires. Many measures had Spanish versions readily available including the Family Adaptability and Cohesion Evaluation Scale—Fourth Edition (FACES-IV; [54]), the Family Communication Scale (FCS; [54]), the Family Satisfaction Scale (FSS; [54]), and Sense of Coherence Scale (SOC-13; [51]). Measures that did not have Spanish versions (i.e., Resilience Scale for Adults (RSA; [55]), Life Orientation Test-Revised (LOT-R; [56]), Relationship-Focused Coping Scale (RFCS; [57]), and the Family Assessment Device—General Functioning (FAD-GF; [58]) utilized Chapman and Carter's [59] methodology for translation. Measures were translated by a bilingual and bicultural researcher in Spanish and then translated back into English by a different bilingual and bicultural researcher. Any inconsistencies between the original English version and the Spanish-to-English were jointly addressed.

#### 2.2.1. Family Adaptability and Cohesion Evaluation Scale—Fourth Edition (FACES-IV)

Family cohesion and flexibility were assessed using the Spanish version of the FACES-IV [60]. Balanced and unbalanced domains of flexibility in the family (e.g., “It is important to follow the rules in our family”) and cohesion (e.g., “Family members seem to avoid contact with each other when at home”) were measured with the current scale. The subscales purport to measure the lower and upper limits of cohesion (e.g., disengagement and enmeshment) and flexibility (e.g., rigid and chaotic) in the family. These constructs are evaluated with two ratio scores that assess the amount of balance versus unbalance within its respective domain with higher ratio scores indicative of healthier or more balanced systems [54]. The Spanish version of the FACES-IV has demonstrated good internal consistency and adequate convergent, concurrent, and content validity (*α* = 0.87; [60]).

#### 2.2.2. The Family Communication Scale (FCS)

The quality of each family's communication patterns was assessed using a Spanish version of the FCS [54]. Total scores for this 10-item measure (e.g., “Family members are very good listeners”) range from 10 to 50 with higher scores indicating better communication. Even though no psychometric data are available for the Spanish version of the FCS, the English version has shown good internal consistency (*α* = 0.97) and test-retest reliability [54].

#### 2.2.3. The Family Satisfaction Scale (FSS)

The extent to which family members are happy, content with each other, and satisfied with their overall family functioning was assessed with a Spanish version of the FSS [54]. This 10-item scale (e.g., “Your family's ability to cope with stress”) assesses participants' level of family satisfaction. The minimum score is 10, and the maximum is 50, with higher scores indicating more satisfaction. The FSS has demonstrated excellent internal consistency (*α* = 0.93) and good discriminant validity [54].

#### 2.2.4. Family Assessment Device—General Functioning (FAD-GF)

General problems within the family structure were assessed using the FAD-GF [58]. This measure is composed of 12 items (e.g., “Planning family activities is difficult because we misunderstand each other”) with higher mean scores indicating more problematic functioning (range: 0 to 4). Since a Spanish version was not readily available, the FAD-GF was translated for the purpose of this study. The English version of the FAD-GF has been shown to have good discriminant validity [54] and internal consistency (*α* = 0.83; [61]); however, the scale evidenced good internal consistency within the current sample (*α* = 0.89).

#### 2.2.5. Relationship-Focused Coping Scale (RFCS)

Familial empathy was assessed using the RFCS that aims to evaluate the protection and maintenance of familial relationships during stressful periods using empathic responses [57]. This 10-item measure is composed of two facets of empathic responding, behavioral (e.g., “Tried to help the other person(s) involved by listening to them”) and cognitive/affective (e.g., “Tried to experience what the other person was feeling”) along a 4-point scale. Total scores range from 0 to 40 with higher scores indicative of higher empathic responding. The scale has been shown to have excellent internal consistency (*α* = 0.93; [57]).

#### 2.2.6. Resilience Scale for Adults (RSA)

The degree to which an individual's protective resources promote resilience was measured using the RSA [55]. Originally, this scale had 45 items and included five dimensions: social support, personal structure, family coherence, personal competence, and social competence. Per the recommendation of the scale authors, a 33-item version was used for the current study [54]. Total scores range from 33 to 231 with greater scores indicative of greater resilience. Adequate reliability has been shown for each subscale with *α*'s ranging from 0.67 to 0.90. The internal consistency for this sample was excellent (*α* = 0.96).

#### 2.2.7. Sense of Coherence Scale (SOC-13)

Sense of coherence, a global view of life as predictable, meaningful, and viewed as a form of resilience or coping, was assessed by the SOC-13 [51]. The 13-item scale covers three dimensions, comprehensibility, manageability, and meaningfulness. Items are rated along a 7-point scale with each item requiring different responses, such as “very often” to “very seldom” and “never happened” to “always happened,” depending on the item. Higher total scores indicate high coping ability and range from 13 to 91. The scale has been validated on eight Spanish samples with satisfactory psychometric properties for Spanish speakers across various ages, genders, levels of functioning, and disability [62].

#### 2.2.8. Life Orientation Test-Revised (LOT-R)

Dispositional optimism was assessed using the 10-item LOT-R [56]. Total scores range between 0 and 40, with higher scores indicating greater optimism and a more positive view on life. This scale has shown satisfactory internal consistency (*α* = 0.78), with additional evidence showing appropriate convergent and divergent validity [56].

### 2.3. Procedure

At the Instituto de Neurociencias de San Lucas in Rosario, Argentina, participants were recruited through routine visits to the attending neurologist. During the appointment, the caregivers were given the measures by a psychologist in approximately one hour. IRB approval from the University of Deusto was received for the study location, and participants from the site fully consented to participate in the study.

## 3. Results

### 3.1. Distribution of Scores

The means for the family dynamics variables appear in Table 2. According to criteria established by the authors of the measures [54], the majority of the caregivers in this sample had connected families (70.5%), some had very connected families (18%), and the others had somewhat connected families (11.5%). The majority of caregivers came from flexible families (88%), some came from somewhat flexible families (10%), and only a few came from very flexible ones (2%). Of the caregivers' families, 6.5% were considered to have very high communication, 26.5% high communication, 26% moderate communication, 13% low communication, and 28% very low communication. On the Family Satisfaction Scale, the majority of the sample exhibited low satisfaction, with 43% of caregivers rating their satisfaction as very low, 20% rating as low, 4.5% as moderate, 25% as high, and only 7.5% as very high. On the Relationship-Focused Coping Scale, the sample's mean (SD) of 1.96 ± 0.53 was higher than the original scale's mean (SD) of 1.64 ± 0.64 [57], which indicates that the level of empathy in this sample was high. On the Family Assessment Device, which measured family problems, 51.4% of the sample was classified as having healthy family functioning and 48.6% as having unhealthy family functioning [58].

### 3.2. Normality Assumptions

Normality assumptions were checked before running the principle statistical analyses. Normality assumptions were met for all variables. Skewness values ranged from −0.577 to 0.010, and kurtosis values ranged from −0.873 to 1.470. As a result, no transformations were needed. Tolerance and VIF values were used to assess multicollinearity. Tolerance values (range: 0.23 to 0.96) were greater than 0.2 and VIF values (range: 1.04 to 4.47) were less than 10 indicating no significant issues with multicollinearity [63].

### 3.3. Correlation Matrix

A correlation matrix (Table 2) was generated to examine the bivariate relationships among all variables in the current study. Empathy, cohesion, flexibility, and communication were found to be negatively correlated with family problems and positively related to all other variables except for optimism. Family satisfaction, resilience, and sense of coherence were also found to be negatively correlated with family problems but were positively correlated with all variables including optimism. Family problems were negatively correlated with all variables at a 0.01 level except for optimism, which was significant at the 0.05 level.

### 3.4. Resilience

Bivariate correlations between caregiver demographics and resilience indicated that only income was significantly related to resilience (*r* = 0.33 and *p* = 0.001) and as a result, income was controlled for in analyses. In the first hierarchical multiple regression (Table 3) with resilience as the dependent variable, income was entered into the first step, which was significant, with *F*(1,103) = 12.18, *p* = 0.001, and *R* ^2^ = 0.11. The second step including all six variables of family dynamics was also significant, with *F*(7, 97) = 10.11, *p* < 0.001, and *R* ^2^ = 0.42, and the amount of variance in resilience that was explained increased significantly, with Δ*F*(6, 97) = 8.84, *p* < 0.001, and Δ*R* ^2^ = 0.32. In the second model, the family dynamics of empathy and family problems were both uniquely associated with resilience with income remaining statistically significant (Table 3). No other family dynamics were significant unique predictors (all *p*s ≥ 0.414).

### 3.5. Sense of Coherence

Bivariate correlations between caregiver demographics and sense of coherence indicated that gender was significantly related to sense of coherence (*r* = 0.23 and *p* = 0.017), such that women caregivers reported higher sense of coherence than men caregivers. Subsequently, further analyses controlled for gender. In the second hierarchical multiple regression (Table 4) with sense of coherence as the dependent variable, gender was entered into the first step, which was significant, with *F*(1, 103) = 5.88, *p* < 0.05, and *R* ^2^ = 0.05. The second step included all the family dynamics variables, which was also significant, with *F*(7, 97) = 11.22, *p* < 0.001, and *R* ^2^ = 0.45, and the amount of variance in sense of coherence that was explained increased significantly, with Δ*F*(6, 97) = 11.22, *p* < 0.001, and Δ*R* ^2^ = 0.39. While gender was significant in the first model, it failed to maintain significance in the second model (*p* = 0.221). Of the family dynamics variables, only family problems and communication were significant, with empathy approaching significance (*p* = 0.067; Table 4). No other family dynamics were significant unique predictors (all *p*s ≥ 0.239).

### 3.6. Optimism

Bivariate correlations between caregiver demographic variables and optimism did not show any significant relationships. Consequently, no demographics were entered as controls in the third regression with optimism as the dependent variable (Table 5). When the six family dynamics were entered as independent variables, the overall model was found to be statistically nonsignificant, with *F*(6, 98) = 1.20, *p* = 0.311, and *R* ^2^ = 0.07, indicating that family dynamics did not together predict optimism in the current sample. Furthermore, no family dynamics were significant unique predictors in the overall model (all *p*s ≥ 0.231).

## 4. Discussion

The purpose of the current study was to examine whether healthier family dynamics were associated with higher resilience, sense of coherence, and optimism among dementia caregivers in Argentina. After controlling for significant demographic and caregiver characteristics, family dynamics explained approximately 32% of the variance in resilience and 39% of the variance in sense of coherence. The primary results of the study found that greater empathy was uniquely associated with greater resilience and was trending in significance with increased sense of coherence. Having few family problems was uniquely associated with greater resilience and increased sense of coherence, while greater communication was independently associated with greater sense of coherence. Optimism was not found to be significantly associated with family dynamics.

The family dynamics in the current sample were generally healthy. Almost two-thirds of caregivers indicated that their families had moderate to very high levels of communication with a vast majority of caregivers rating their families as connected and flexible. Such broad healthy family dynamics may be due to the importance of* familismo* imbedded in the Latino culture that values having stronger family values. This sample, however, was found to be generally less satisfied with their family dynamics. This could be due to the measure of family satisfaction being originally created without cultural sensitivity for familism. It is also likely that because our sample comes from a culture with a high emphasis on family they held higher standards of family ideals and how their families should function.

The overall pattern of findings from the study is consistent with previous literature in which caregivers from families with overall healthier dynamics are more likely to have greater personal strengths through better quality of life. Individuals from families with poor functioning are likely to experience increased caregiver burden [19], which is associated with decreased coping [40]. Research on the origin of psychological strengths has emphasized the role of* sense of coherence* and* general resistance resources* (similar to resilience) in the face of stressors and suggests the importance of social support and expressions of support in increasing sense of coherence and coping abilities. This is consistent with the findings of the current study that sense of coherence and resilience are associated with environmental factors such as family dynamics.

One family factor that was identified as particularly important for the development of caregiver personal strength is empathy, which partially supported our hypothesis. These results are consistent with prior research on resilience that has identified increased empathy as a protective factor in dealing with stressors [64] and may thus promote greater adaptive coping and facilitate resilience. By extension, empathy has been derived as a core component for benefit finding among caregivers for individuals with cancer [65], suggesting that caregivers of people with long-term illnesses who are more empathic may be more likely to derive meaning from the caregiving experience and view life as more meaningful. This could likely promote more adaptive coping and increased resilience.

Family pathology was also identified as being particularly important for resilience and sense of coherence, which generally supports the study hypothesis. Previous research on the salutogenic nature of sense of coherence in the face of stressors is consistent with the current study's finding connecting family problems and sense of coherence [53]. In a family with increased family problems, it may be difficult for an individual to cope with stressors, especially in cultures of familism such as in Latin America. Conversely, caregivers who have little family conflict may be likely to perceive stronger family relationships, which is a strong influence on resilience [66]. Since the family is a primary source of social support and identity, an individual from a family high in conflict is likely to have difficulties coping with the daily stressors of caregiving. While sense of coherence may help individuals cope with a stressful family environment, a supportive environment is also likely to help an individual better cope with personal stressors and promote resilience.

Though less consistent across the different personal strengths and in partial support of the study hypothesis, family communication was uniquely associated with sense of coherence. Prior work with adolescents has found that those who found it easier to communicate with their parents had greater sense of coherence [52]. Indeed, maintaining satisfactory family communication facilitates adaptive coping within the family system and promotes individual growth and development [67]. This may play a unique role among caregivers as many may view the caregiving role as an opportunity for personal growth, especially as the disease progresses and the individual with dementia becomes increasingly more dependent on the caregiver. The role of familism in this population may additionally direct the caregiver to view their caregiving role as an opportunity to show family loyalty and reciprocity, bolstering the positive effects of healthy family communication and viewing life as meaningful.

When examining family dynamics regressed onto optimism, the results were nonsignificant. These findings may be explained by the nature of optimism as a character trait rather than one that is influenced by external factors. Studies on caregivers of children with cancer have failed to find relationships between optimism and environmental factors such as cancer severity, suggesting the trait-like nature of optimism and that environmental factors like family dynamics are not expected to play a major role in increasing optimism [68]. Other studies also have shown that levels of optimism vary by nation, which suggests that culture, rather than family, may have a greater influence on individual optimism [69].

### 4.1. Limitations

This study has several limitations and directions for future research. First, this study does not establish causation or temporal inference because of its cross-sectional design. For these reasons, causal and temporal inferences should be established in future studies exploring the association between family dynamics and personal strengths over time using cross-lagged panel designs, latent growth models, or growth mixture models. Secondly, the self-report nature of the measures may have contributed to participant response bias. Participants with heightened caregiver depression or greater optimism, for example, could have over- or underexaggerated their assessments of family dynamics. Future research should include more objective reports of family dynamics by other family members or by trained professionals. Thirdly, the study sample was only comprised of caregivers from Argentina indicating that participants may not be representative of the diverse Latin American population including noncaregivers. Consequently, future studies should examine the relationship between family dynamics and personal strengths in participants from a diverse range of countries and cities in Latin America as well as among noncaregiving samples. Lastly, while the focus of the study was on examining the general pattern of relationships between family dynamics and personal strengths, we did not test any potential moderators or mediators of any of the proposed relationships. It is possible that family dynamics and personal strengths fit within larger and more complex theoretical family system models such as structural family theory [70] applied to caregivers. Thus, future work with sample sizes larger than 200 participants could test these models more directly using different statistical analyses such as structural equation modeling.

### 4.2. Future Directions

The findings of the current study can inform future research on the role of key family dynamics in caregiver personal strengths among understudied and collectivist populations. The family environments in which dementia caregivers function may play an important role in the development and maintenance of personal strengths. Additionally, caregiver personal strengths act as protective factors in the face of daily stressors associated with caregiving, and cultivating these strengths could have a ripple effect on the family environment in which these individuals function. Incorporating family systems interventions targeting communication, empathy, and family problems may enhance personal strengths in caregivers and allow them to provide a higher quality of care for individuals with dementia.

## 5. Conclusion

This study demonstrated that family dynamics were associated with personal strengths in an Argentinian sample of dementia caregivers. In general, greater empathy, more communication, and fewer family problems were unique predictors of greater personal strengths suggesting that healthier family dynamics may be important for caregivers with strong family values. While the findings of this study are preliminary, they in part do suggest that they may be important for informing family systems intervention research for dementia caregivers particularly on increasing empathy and communication and decreasing family problems in the family system. Doing so may improve quality of care for individuals with dementia through greater personal strengths in caregivers.

## Figures and Tables

**Table 1:** Caregiver sociodemographic characteristics.

Sociodemographic characteristic	Family dementia caregivers (*N* = 105)
Gender, % female	74.3
Caregiver marital status, %	
Married	75.2
Single	9.5
Divorced/separated	5.8
Other	9.6
Education, %	
Elementary/primary	18.1
Some high school	1.9
Completed high school	43.8
Technical studies	1.9
Some college	1.9
Completed college	32.4
Socioeconomic level, %	
1-2 (times the minimum wage)	10.5
2-3	44.8
3-4	29.5
4+	15.2
Caregiver age, M (SD)	57.71 (13.35)
Period of care in months, M (SD)	48.21 (23.13)
Hours caregiving per week, M (SD)	63.54 (18.55)

*Note*. M = mean; SD = standard deviation.

**Table 2:** Correlations between family dynamics and personal strengths.

	1	2	3	4	5	6	7	8	9	Mean (SD)
(1) Empathy	—									
(2) Problems	−0.30^b^	—								2.09 (0.48)
(3) Cohesion	0.32^b^	−0.67^b^	—							2.44 (1.17)
(4) Flexibility	0.22^a^	−0.52^b^	0.79^b^	—						1.82 (0.62)
(5) Communication	0.33^b^	−0.79^b^	0.65^b^	0.54^b^	—					34.45 (7.86)
(6) Family satisfaction	0.25^b^	−0.78^b^	0.56^b^	0.40^b^	0.83^b^	—				30.75 (9.72)
(7) Resilience	0.33^b^	−0.53^b^	0.38^b^	0.22^a^	0.40^b^	0.42^b^	—			204.29 (21.28)
(8) Sense of coherence	0.35^b^	−0.60^b^	0.41^b^	0.31^b^	0.59^b^	0.48^b^	0.43^b^	—		68.67 (10.27)
(9) Optimism	0.07	−0.23^a^	0.15	0.04	0.17	0.20^a^	0.51^b^	0.27^b^	—	16.98 (3.00)

*Note*. ^a^Correlation is significant at the 0.05 level (2-tailed). ^b^Correlation is significant at the 0.01 level (2-tailed).

**Table 3:** Hierarchical multiple regression associations between caregiver income, family dynamics factors, and resilience.

Variable	Model 1			Model 2		
	*B*	SE *B*	*β*	*B*	SE *B*	*β*
Income	7.88	2.26	0.33^c^	7.63	1.91	0.32^c^
Empathy				0.72	0.34	0.18^a^
Family problems				−20.48	6.39	−0.46^b^
Cohesion				2.20	2.69	0.12
Flexibility				−3.05	4.48	−0.09
Communication				−0.24	0.44	−0.09
Family satisfaction				0.14	0.33	0.06
*R* ^2^		0.11			0.42	
Δ*R* ^2^		0.11			0.32	
*F* for change in *R* ^2^		12.12			8.84	

*Note*. SE = standard error.

^a^ *p* < 0.05, ^b^ *p* < 0.01, and ^c^ *p* < 0.001.

**Table 4:** Hierarchical multiple regression associations between caregiver income, family dynamics factors, and sense of coherence.

Variable	Model 1			Model 2		
	*B*	SE *B*	*β*	*B*	SE *B*	*β*
Gender	5.43	2.24	0.23^a^	2.27	1.85	0.10
Empathy				0.30	0.16	0.15
Family problems				−9.01	3.05	−0.42^b^
Cohesion				−0.45	1.27	−0.05
Flexibility				−1.23	2.09	−0.07
Communication				0.55	0.21	0.42^a^
Family satisfaction				−0.19	0.16	−0.18
*R* ^2^		0.05			0.45	
Δ*R* ^2^		0.05			0.39	
*F* for change in *R* ^2^		5.88^a^			11.22^c^	

*Note*. SE = standard error.

^a^ *p* < 0.05, ^b^ *p* < 0.01, and ^c^ *p* < 0.001.

**Table 5:** Hierarchical multiple regression associations between caregiver income, family dynamics factors, and optimism.

Variable	Model 1		
	*B*	SE *B*	*β*
Empathy	0.00	0.06	0.00
Family problems	−1.37	1.14	−0.22
Cohesion	0.38	0.48	0.15
Flexibility	−0.91	0.79	−0.19
Communication	−0.02	0.08	−0.04
Family satisfaction	0.02	0.06	0.06
*R* ^2^		0.07	

*Note*. SE = standard error.

## Additional Points

Although the findings from this study are preliminary, they do suggest areas that may be important for clinical research targeting families of individuals with dementia in Latin America and, especially, in Argentina. Given the significant associations of increased family empathy and decreased family problems with caregiver resilience, empathy-increasing intervention research and those designed to decrease pathological responding within the family system could have the potential to increase caregiver resilience in families of individuals with dementia. Similarly, the findings on the association between family communication and family problems with caregiver sense of coherence could also inform research on family systems interventions targeting families of individuals with dementia. Previous studies have already shown the importance of family communication interventions for both caregivers and individuals with dementia [24]. These types of interventions also likely have the potential to increase caregiver sense of coherence, though this hypothesis awaits empirical support. It is reasonable that fewer family problems and better family communication would contribute to a healthier environment for the caregiver as he or she feels more in control of the situation, especially since meaningfulness and manageability are both aspects of sense of coherence.
